# Microgravity promotes osteoclast activity in medaka fish reared at the international space station

**DOI:** 10.1038/srep14172

**Published:** 2015-09-21

**Authors:** Masahiro Chatani, Akiko Mantoku, Kazuhiro Takeyama, Dawud Abduweli, Yasutaka Sugamori, Kazuhiro Aoki, Keiichi Ohya, Hiromi Suzuki, Satoko Uchida, Toru Sakimura, Yasushi Kono, Fumiaki Tanigaki, Masaki Shirakawa, Yoshiro Takano, Akira Kudo

**Affiliations:** 1 Graduate School of Bioscience and Biotechnology, Tokyo Institute of Technology, Yokohama 226-8501, Japan; 2 Section of Biostructural Science, Graduate School of Medical and Dental Sciences, Tokyo Medical and Dental University, Tokyo 113-8549, Japan; 3 Section of Pharmacology, Department of Bio-Matrix, Graduate School, Tokyo Medical and Dental University, Tokyo 113-8549, Japan; 4 Department of Science and Applications, Japan Space Forum, Tsukuba 305-8505, Japan; 5 Mitsubishi Heavy Industries, Ltd. Kobe 652-8585, Japan; 6 Japan Aerospace Exploration Agency, Tsukuba 305-8505, Japan

## Abstract

The bone mineral density (BMD) of astronauts decreases specifically in the weight-bearing sites during spaceflight. It seems that osteoclasts would be affected by a change in gravity; however, the molecular mechanism involved remains unclear. Here, we show that the mineral density of the pharyngeal bone and teeth region of TRAP-GFP/Osterix-DsRed double transgenic medaka fish was decreased and that osteoclasts were activated when the fish were reared for 56 days at the international space station. In addition, electron microscopy observation revealed a low degree of roundness of mitochondria in osteoclasts. In the whole transcriptome analysis, *fkbp5* and *ddit4* genes were strongly up-regulated in the flight group. The fish were filmed for abnormal behavior; and, interestingly, the medaka tended to become motionless in the late stage of exposure. These results reveal impaired physiological function with a change in mechanical force under microgravity, which impairment was accompanied by osteoclast activation.

It is considered that gravity is a key factor playing roles in the tissue construction^1^ and remodeling of bone^2^,^3^. The nature of the microgravity environment has led us to assume that alteration of external forces leads to imbalance of static sense, cell activity, and physiology. In fact, the BMD of astronauts is known to decrease^3^,^4^, suggesting that osteoclasts and osteoblasts would be affected by a change in gravity^5^. Recently, it was reported that bisphosphonate treatment coupled with exercise protected against bone loss in humans during long-duration spaceflight^6^, indicating that osteoclasts are activated under microgravity. However, the mechanisms are unclear; and most *in vivo* studies did not show clear changes in osteoclast number or activity during spaceflight^7^.

In this present study, to examine the cellular activities related to bone formation and resorption in a microgravity environment during animal growth, we utilized the growth of bones of transgenic medaka fish from the juvenile to adult stage in an experiment conducted in space. We mainly examined the pharyngeal region where many osteoclasts are found^8^, which region contains several hundreds of teeth in the adult fish and is highly sensitive to gravity because of this high density of teeth in this area.

The fish were filmed for abnormal behavior during their 60 days of being systematically reared under microgravity. We found that the mineral density of the pharyngeal bone and teeth of the medaka fish decreased and that osteoclasts were activated when the fish were reared for 56 days at the international space station (ISS). In the whole transcriptome analysis of jaw tissue from medaka reared for 60 days at ISS, several genes in the downstream of the glucocorticoid receptor (GR)^9^ were strongly up-regulated in the flight group. In addition, we found enhanced expression of 15 mitochondrion-related genes; and electron microscopy observation revealed a low roundness of mitochondria in the osteoclasts. Thus, our study provides a new insight regarding the regulation of bone loss under microgravity.

## Results

### Generation of osteoclast- and osteoblast-reporter fish

Firstly, we established TRAP promoter-GFP/Osterix promoter-Dsred double transgenic medaka fish for this experiment, because osteoclasts and osteoblasts in addition to tooth buds in the pharyngeal region are divided by 2 fluorescent signals in this transgenic line. Twenty-four candidate fish of 16 mm in total length (6 weeks after hatching) were selected from 312 fish by 3 criteria: no sickness, same size with growth and good vision. These 24 candidate fish were transported into space and reared for 2 months in the AQH (Aquatic Habitat) in the “Kibo” section of the ISS. RNAs were isolated from 8 fish at day 0, and from 4 fish at day 60, after the start of rearing in the AQH; and 6 fish were fixed with PFA at day 14 and 6 fish at day 56 by the help of astronauts in the ISS (Supplementary Table 1).

### Medaka fish behaviors under microgravity

**Figure 1:**
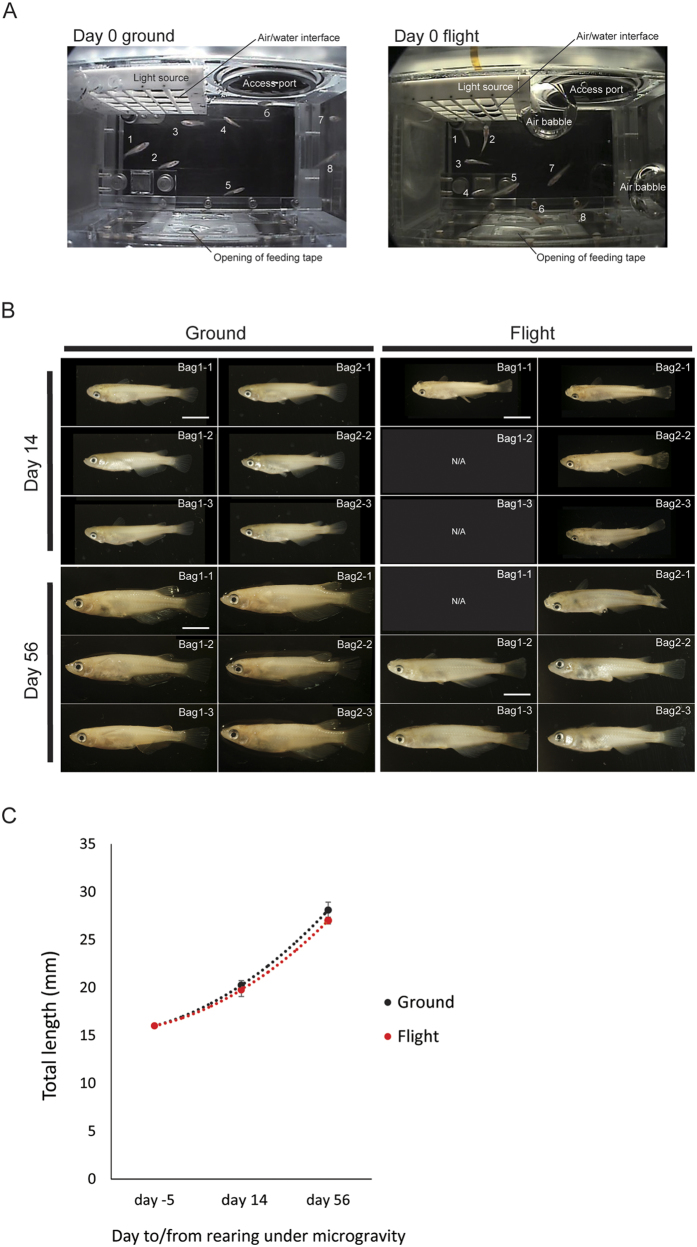
Rearing and PFA fixation of the ground and flight TRAP-GFP/Osterix-DsRed transgenic medaka fish. (**A**) Appearance of reared fish in AQH of the ground control (left) and the spaceflight (right) at day 0. Light source, Air/water interface and Access port are localized at the upper side, and the feeding place with an automatic feeder is located at the lower side in this figure. The 8 numbers shows the locations of 8 fish in a given aquarium. The air babbles are indicated only in the flight-group AQH. (**B**) A whole view of PFA-fixed fish. The ground control at day 14 after launch (upper left, 6 fish), the flight group at day 14 (upper right, 4 fish), the ground control at day 56 (lower left, 6 fish) and the flight at day 56 (lower right, 5 fish) are shown. Three fish were not available (N/A). Scale bars show 5 mm. (**C**) Graph showing the total length for the ground control medaka (black dotted line) and the flight medaka (red dotted line) at 3 days before launch, and 14 days and 56 days after the start of experiment. Bars represent the mean ± SD.

Unique behaviors of the medaka fish in the AQH were filmed during 2 months to observe their adaptation to the microgravity environment (Fig. 1A). Observation at day 0, immediately after transport to the AQH, showed that the fish swam gently and seldom displayed “looping behavior,” i.e., swimming in tight circles, indicating that the fish had the capacity to adapt to the microgravity environment (Supplementary Movie 1). At day 14, before being fixed with PFA the fish swam vertically and looped, showing an upside-down behavior that seemed to indicate that they were trying to assume an appropriate posture under microgravity for eating or swimming (Supplementary Movie 2). At day 27, the fish could be induced to mate successfully under microgravity (Supplementary Movie 3). Interestingly, the medaka tended to become motionless at day 47, suggesting that they behaved like animals having hypokinesia (Supplementary Movie 4). These movies demonstrated specified unusual behaviors for adaptation to microgravity, suggesting that physiological changes had occurred in the flight fish just as in astronauts.

### Flight fish group shows normal growth

For examination of bone tissues, medaka fish were fixed with 4% paraformaldehyde at days 14 and 56 (Fig. 1B). The measurement of total length (5 days before rearing and 14 days and 56 days after the start of rearing) showed the same growth tendency between the ground and flight groups (Fig. 1C). With respect to the development of pharyngeal teeth, there was a slightly lower number of teeth at day 14, but almost the same number at day 56, in the microgravity-exposed medaka than in the ground group (Supplementary Fig. 1), suggesting that tooth development in terms of tooth germ formation was normal in the flight group.

### Mineral density was decreased during 56 days under microgravity

**Figure 2:**
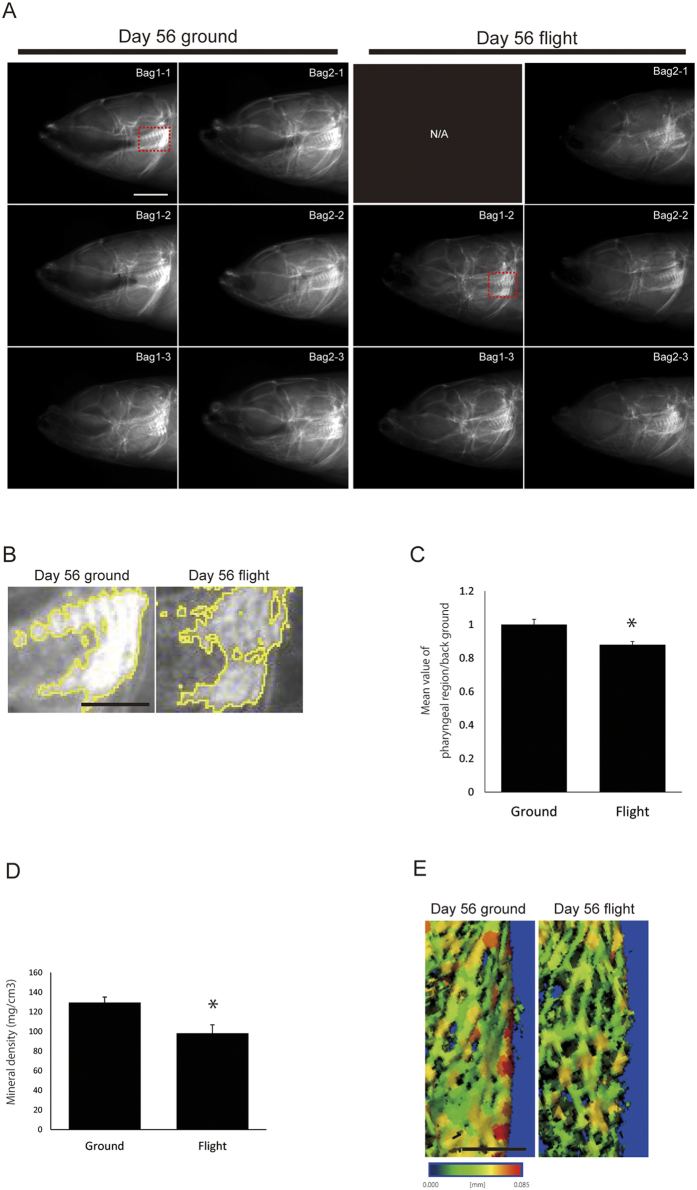
Mineralization status of the pharyngeal bone at day 56. (**A**) Soft X-ray analysis. Lateral views of the heads of ground (left) and flight medaka (right) reared for 56 days. The red-dotted box in (Bag1-1 ground and Bag1-2 flight) shows the pharyngeal bone region depicted in “B.” Scale bar indicates 1 mm. (**B**) Magnified view of the red-dotted box in “A.” The mineralized area (white) was selected and outlined in yellow by using “ImageJ” software. Scale bar indicates 500 μm. The left-hand side of each figure is rostral. (**C**) Comparison of “mean gray value” as the mineralized signals at the pharyngeal region in the ground and flight medaka, normalized with the value of the background. *P < 0.05, by Student’s *t*-test; ground n = 6, flight n = 5. Error bars, s.e.m. (**D**) Mineral density of pharyngeal bone measured by pQCT. *P < 0.05, by Student’s *t*-test; ground n = 6, flight n = 5. Error bars, s.e.m. (**E**) Graded-color images for focal average thickness of the ventral inner part of the upper pharyngeal bone. Thicker places are shown in red; and thinner ones, in green. The color bar shows the thickness parameter in which red color indicates 0.085 mm and blue color, 0 mm. Scale bar represents 100 μm.

For evaluation of mineralization under microgravity, soft X-ray analysis of whole fish was performed (Fig. 2A). In the flight fish group at day 56, the calcified area (white signals) in the pharyngeal bone region was decreased, compared with that for the ground control (Fig. 2B,C); whereas there was no significant difference in calcification at day14 (Supplementary Fig. 2). The mineral density of the upper pharyngeal bone and the tooth region showed an approx. 24% decrease in the pQCT analysis, compared with that for the ground control (Fig. 2D; ground control: 129.417 ± 13.904 mg/cm^3^ n = 6, flight: 98.14 ± 19.265 mg/cm^3^ n = 5, p < 0.05). In addition, μCT imaging showed thinner bone in the flight medaka (Fig. 2E). Regarding the vertebral bones, their BMD was slightly decreased (Supplementary Fig. 3). In addition, in another calcified area, the BMD of otoliths showed no significant difference between the flight and the control samples (data not shown).

### Osteoclast volume was increased during 56-day flight

**Figure 3:**
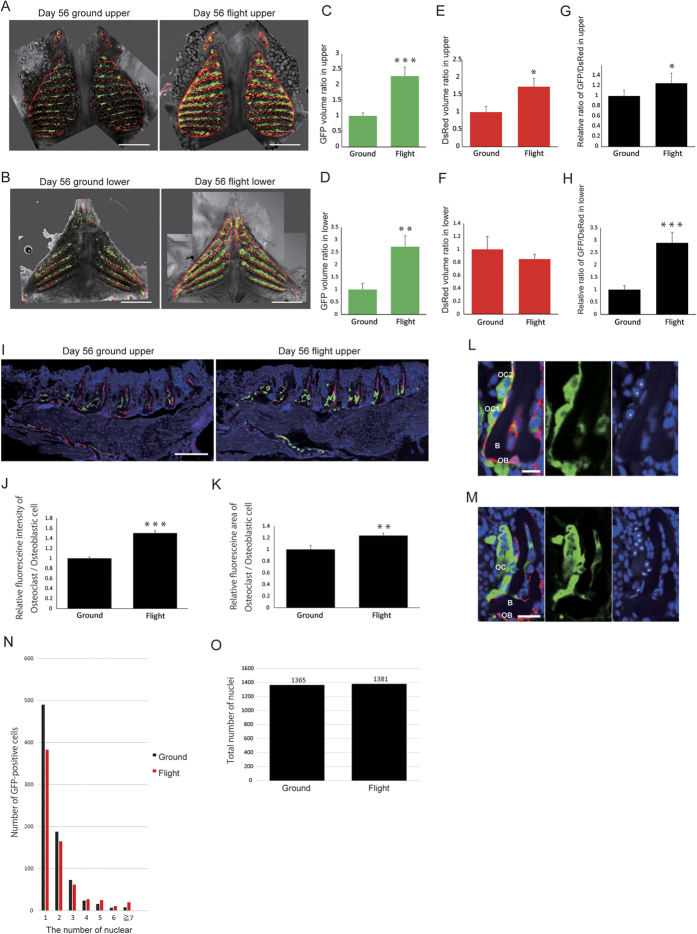
Fluorescence imaging of pharyngeal bone shows enlargement of osteoclasts in the flight medaka at day 56. (**A**,**B**) Upper (**A**) and lower (**B**) pharyngeal bones and teeth at day 56. Bars represent 400 μm. Top of figures indicates rostral. (**C**–**F**) Quantitative analysis of the GFP and DsRed volumes derived from fluorescent signals in the upper (**C**,**E**) and lower (**D**,**F**) pharyngeal bone. Upper pharyngeal bone: ground control n = 12, flight n = 10; lower pharyngeal bone: ground control n = 12, flight n = 9. (**G**,**H**) Graphs show the relative value of GFP volume divided by DsRed volume. (**I)** Double antibody-staining of paraffin sections of upper pharyngeal bones and teeth with anti-GFP and anti-DsRed antibodies. Anti-GFP antibody was labeled with FITC, and anti-DsRed was detected by Cy-3-conjugated second antibody. Nuclei were stained with DAPI. The left-hand side is rostral. Scale bar indicates 100 μm. (**J**) Comparison between the ground and flight groups with respect to the signal intensity of GFP per DsRed. (**K**) Comparison between the ground and flight regarding the GFP-positive area per DsRed area. (**L**) Enlarged images of typical mononuclear cell and a 2-nuclei cell. One GFP-positive cell (OC1) contains 1 nucleus (asterisk); and another, 2 nuclei (asterisks, OC2). Merge (left), GFP (middle), and DAPI (right). OC; osteoclast, OB; osteoblast, B; bone. Scale bar indicates 10 μm. (**M**) A typical multinucleate GFP-positive cell (OC) containing 10 nuclei (asterisks). Merge (left; GFP, DsRed, and DAPI), GFP (middle), and DAPI (right). OC, osteoclast; OB, osteoblast; B, bone. Scale bar represents 10 μm. (**N**) Graph comparing ground and flight groups regarding numbers of GFP-positive cells including each of the indicated number of nuclei. Data are derived from “Supplementary Table 2.” (**O**) Comparison between the ground and flight groups as to the total number of nuclei in GFP-positive cells. (**I**–**O**) GFP- and DsRed-positive areas in 30 serial paraffin sections of pharyngeal bone region were selected from ground medaka (n = 3) and flight medaka (n = 3) at day 56. *P < 0.05, **P < 0.005, ***P < 0.0005, by Student’s *t*-test. Error bars, s.e.m.

Since TRAP is a marker of osteoclasts and osterix is a marker of osteoblasts, the whole upper and lower pharyngeal bones at day 56 were imaged by confocal laser scanning microscopy (Fig. 3A,B); and the volumes of fluorescent TRAP-GFP and Osterix-DsRed were measured by use of software, because the cell volume is an important parameter for resorption capacity in addition to the surface area of resorption, according to theoretical calculation of cell fusion and bone resorptive capacity^10^. According to the quantitative analysis of GFP (Fig. 3C,D) and DsRed (Fig. 3E,F) volumes, the GFP volume was particularly increased in the flight group. The osteoclast volume was normalized by the osteoblast volume, because the sizes of pharyngeal bones varied, with the result being that the relative ratio of the GFP volume divided by the DsRed volume was increased during flight (Fig. 3G,H). This finding indicated that the osteoclast volume in the flight medaka had increased over that of the osterix-expressing osteoblastic cells. However, fluorescent imaging of the vertebral body showed only a slight but no significant enlargement of osteoclasts (Supplementary Fig. 4). Next, to examine the fluorescence intensity and the area of osteoclasts and osteoblastic cells in the upper pharyngeal bone, we performed double immunostaining by using anti-GFP and anti-DsRed antibodies (Fig. 3I). The relative fluorescence intensity calculated from the ratio of osteoclast signals divided by osteoblastic signals in the flight group at day 56 was increased by 50.4% over that for the ground group (Fig. 3J), and the relative area of fluorescence calculated from the osteoclast area divided by osteoblastic signals in the flight group was increased by 23.8% by over that of the ground group (Fig. 3K). On the other hand, only the fluorescence intensity in the flight group was increased at day 14 (Supplementary Fig. 5). Whole 3D reconstruction of serial sections indicated the enhancement of GFP signals in the flight group of medaka (Supplementary Movie 5). Because osteoclasts become mature by cell fusion, we determined the numbers of several types of GFP-positive osteoclasts having different numbers of nuclei, e.g., 1 nucleus (mononuclear), 2 nuclei per cell (Fig. 3L), 10 nuclei per cell (Fig. 3M), etc. and counted the number of TRAP-GFP-positive cells containing each of these numbers of nuclei (Fig. 3N and Supplementary Table 2). This result indicated that multi-nucleation was increased under microgravity, although the total number of nuclei showed no significant difference between the ground and flight groups (Fig. 3O). 3D imaging showed enhancement of osteoclast fluorescent signals throughout the whole area of upper and lower pharyngeal bones in the flight group (Supplementary Movie 6).

### The flight group showed an increased area of TRAP staining and mitochondrial abnormality in osteoclasts

**Figure 4:**
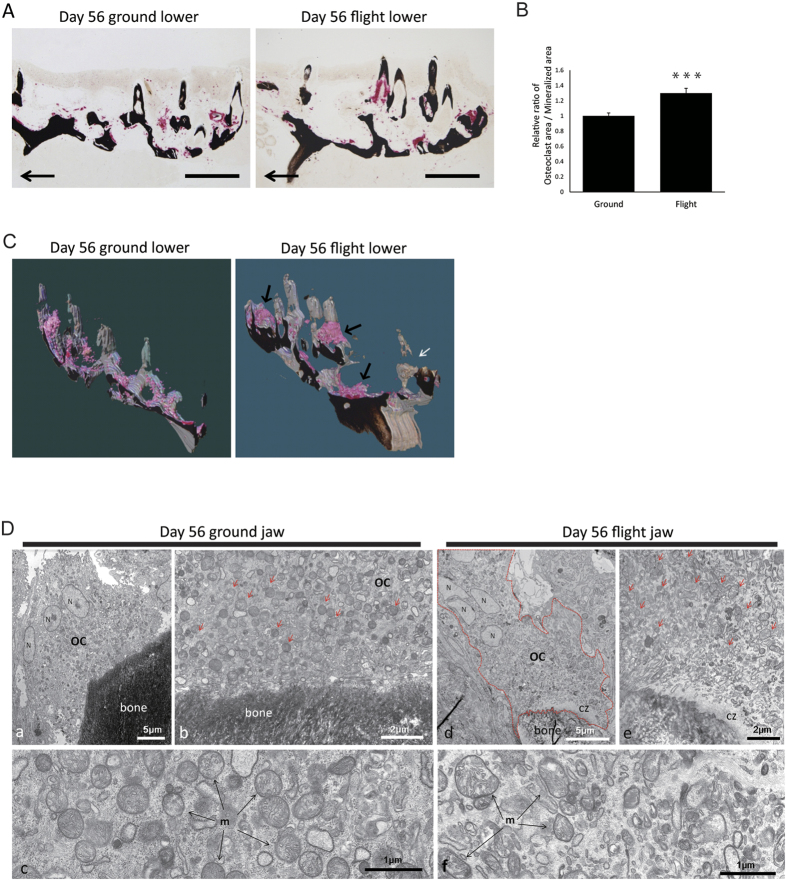
Increase in the TRAP-positive area and change in the mitochondrial structure in the flight group. (**A**) Histochemical demonstration of mineralized matrices (black area) and osteoclasts (red area) in the lower pharyngeal bones and teeth of ground medaka (left) and flight medaka (right) at day 56, as demonstrated by von Kossa/TRAP double staining. Arrows indicate the rostral direction; and scale bars, 200 μm. (**B**) Percent ratio of TRAP-positive osteoclast area relative to von Kossa-positive mineralized area of pharyngeal bones and teeth in randomly selected sections (9 or 10 sections per medaka) prepared from ground (n = 3) and flight fish (n = 3) as shown in “A” and “C.” Proportion of osteoclast area relative to mineralized area was significantly increased in the flight-group medaka at day 56. ***P < 0.0005, by Student’s *t*-test. Error bars, s.e.m. (**C**) Partial 3D models of pharyngeal bones/teeth and osteoclasts from the ground (left) and flight (right) groups at day 56 reconstructed from 4-μm-thick Technovit sections that had been doubly stained for von Kossa and TRAP reactions. The black arrows show large osteoclasts in the flight group. The white arrow indicates a gap in the von Kossa staining due to possible dissolution of minerals from bones and teeth in the flight-group medaka. (**D**) Low degree of roundness of mitochondria in the flight group. (**a**–**c**) Day-56 jaw from the ground group. This multinucleate osteoclast (OC) appears to be devoid of its ruffled border (**a**). High magnification of an osteoclast shows numerous mitochondria in its cytoplasm (**b**), and an even more enlarged view reveals their round shape (**c**). (**d**–**f**) Day-56 jaw from the flight group. Low-power view of a gigantic multinucleate osteoclast (OC), with the cell contour outlined in red, is shown (**d**). A clear zone-like structure (CZ) of an osteoclast attached to bone is evident (**e**). An enlarged view of mitochondria with heterogeneous shapes and sizes (**f**).

Next, to detect the enzymatic activity responsible for bone resorption, we performed TRAP staining of the lower pharyngeal bone at day 14 (Supplementary Fig. 6A–H). The TRAP-positive area expressed as unit area was increased in the flight group (Supplementary Fig. 6I). 3D imaging of TRAP-stained specimens showed dynamic bone resorption in the flight group (Supplementary Fig. 7). Furthermore, lower pharyngeal bones at day 56 were sectioned and stained for TRAP activity and with Von Kossa to examine the proportion of bone resorption per area of bone mineralization (Fig. 4A). The result showed an increase in this relative ratio (Fig. 4B); and 3D imaging of the flight group revealed an accumulation of TRAP-positive areas, indicating that large osteoclasts were present there (Fig. 4C; Supplementary Movie 7). This finding was consistent with the increased area of GFP-positive cells in the lower pharyngeal bone of the flight group found by 3D imaging (Supplementary Fig. 8). Consequently, a gap in the von Kossa staining was found, suggesting possible dissolution of minerals from the bones (Fig. 4C). Next, to examine the morphological change in organelles of osteoclasts, we performed electron microscopic analysis. Our results showed that the mitochondria in osteoclasts showed a low degree of roundness in the jaw of flight-group (Fig. 4D and Supplementary Fig. 9).

### Osteoblastic activity tended to decrease in the flight group

For study of the molecular mechanism of bone loss during the flight period, RNAs were extracted from the medaka jaw at day 60; and transcriptome analysis after cDNA sequencing was performed; because, unfortunately, RNAs extracted from the pharyngeal bone appeared to have undergone degradation because it was difficult to preserve tissues at the back side of the throat by using RNAlater. Bones in the jaw as well as teeth were remodeled by osteoclasts and osteoblasts, similar to the remodeling in the pharyngeal bone (Supplementary Fig. 10). According to our results, the expression of osteoblastic genes including those for *osterix* and *type 1 collagen* was decreased (Supplementary Table 3). Consistently, azan staining showed a tendency for a decrease in the amount of collagen in the matrix in the flight group (Supplementary Fig. 11), although osteoblast morphology examined by electron microscopy showed no difference between the ground- and flight-group medaka (Supplementary Fig. 12). In addition, the expression levels of *RANKL* and *NFATc1* were not significantly increased (data not shown), suggesting that osteoclasts in the flight-group medaka had not been activated with the support of osteoblasts.

### Signals under shear stress-like force were increased in day 56 flight group

**Figure 5:**
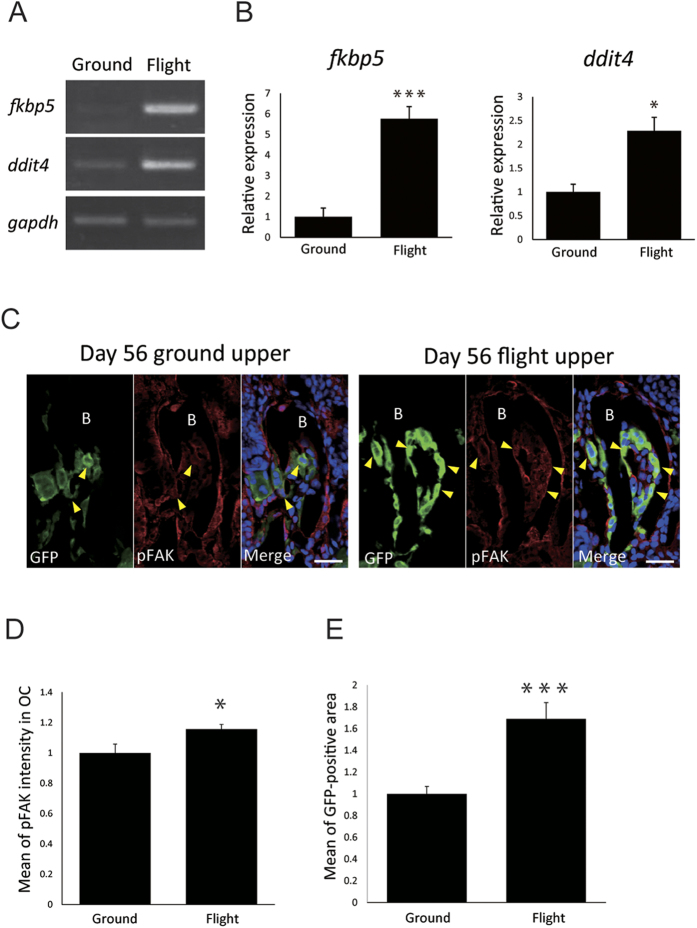
Changes in the transcription level and the phosphorylation state in microgravity. (**A**,**B**) RT-PCR analysis revealed 5.7 fold and 2.3 fold up-regulation of *fkbp5* and *ddit4*, respectively, in the downstream of the glucocorticoid receptor in the flight group. (**A**) Expression of *fkbp5, ddit4*, and *gapdh* mRNA in the ground control and flight medaka. The same data in an uncropped full-length gel is shown in Supplementary Fig. 13. (**B**) Comparison of the relative expression levels of *fkbp5* and *ddit4*, normalized by *gapdh* expression levels. *P < 0.05, ***P < 0.0005, by Student’s *t*-test. Error bars, s.e.m. (**C**–**E**) Comparison of pFAK expression in osteoclasts. (**C**) Double staining with both anti-pFAK and anti-GFP antibodies together with DAPI staining. Scale bars indicate 20 μm. (**D**,**E**) Mean of pFAK intensity “D” and of GFP-positive area “E” was increased in the flight group. The pFAK intensity was normalized by dividing by the GFP-positive area. Five serial sections per medaka were measured in ground (n = 3) and flight (n = 3) group fish. *P < 0.05, ***P < 0.0005, by Student’s *t*-test. Error bars, s.e.m.

To find influence of the abnormal mitochondria structure in osteoclasts, we examined mitochondrion-related molecules including those of the glucocorticoid receptor (GR) pathway^11^,^12^. In the transcriptome sequencing, *fkbp5* and *ddit4* were found to be the genes with the highest elevation at the transcriptional level during flight; and, interestingly, *fkbp5* was strongly and broadly induced in many organs such as scales, a part of the trunk, brain, liver, ovary, and intestines (Dr. Mitani, personal communication) as well as in the jaw in the flight group, which elevated transcription was confirmed by RT-PCR analyses (Supplementary Table 3 and Fig. 5A,B). *fkbp5* and *ddit4* are known as molecules in the downstream of the GR^9^, and it is reported that GR is activated by shear stress^13^. Since phosphorylation of FAK is up-regulated by shear stress^14^, we examined its phosphorylation state in osteoclasts by using anti-phosphorylated FAK (pFAK) and determined the osteoclast area with anti-GFP antibodies (Fig. 5C). pFAK signals were normalized by dividing by the GFP-positive area (Fig. 5D,E), and the results indicated enhanced pFAK signaling during the flight period. This result suggested that microgravity generated stress in the flight group. In addition, 15 mitochondrion-related genes were significantly up-regulated at least 1.2-fold compared with their expression in the ground controls (P < 0.05; Supplementary Table 3); and also the expression of 5 osteoclast-related genes, i.e., *cebpb*, *fosl1*, *fosB*, *c-fos*, and *junb-b* was increased at least 1.5-fold compared with that for the ground controls (P < 0.05; Supplementary Table 3). These results indicated enhanced mitochondrial biogenesis in the activated osteoclasts in the flight-group medaka.

## Discussion

We investigated alteration of osteoclast potency between the flight medaka group and the ground control in terms of osteoclast volume calculated from the green fluorescent signals in osteoclasts, anti-GFP antibody-positive area by immunostaining, and enzymatic activity by TRAP staining. All 3 of these methods revealed osteoclast activation in the pharyngeal bone region. The number of multinucleate osteoclasts was increased in the flight medaka at day 56, whereas the total number of nuclei in GFP-positive cells showed no significant difference, indicating that osteoclast differentiation was not enhanced but that the osteoclast fusion process was promoted under microgravity during the 56-day period. These results showed the activation of osteoclasts that were already localized at bone surface in spaceflight, consistent with the report that the BMD of astronauts decreases specifically in the weight-bearing sites, more local remodeling sites regulated by osteoclasts^3^.

Although there is a report showing mitochondrial alteration in a human lymphocyte cell line during spaceflight^15^, no results indicating such an effect *in vivo* or in osteoclasts have been reported. Presently we found mitochondrial abnormalities of jaw tissues in the flight group, which abnormalities included up-regulation of mitochondrial genes and a low degree of mitochondrial roundness, as a change in organelle morphology, in osteoclasts. Recently, it was reported that ATP in osteoclast mitochondria coordinately regulates osteoclast survival and bone resorption^16^. Consistently, the phenotype of the flight group suggested that osteoclast activation might be regulated by mitochondrial reaction under microgravity. As previously reported, spaceflight leads to a decrease in the number and activity of osteoblasts^17^. In our results, osteoblasts were also slightly inactivated; although no significant changes in the roundness of osteoblast mitochondria were observed (data not shown), indicating that osteoclasts were more affected than osteoblasts by a change in gravity.

In the transcriptome analysis, we found up-regulation of *fkbp5* and *ddit4* genes in the downstream of the glucocorticoid receptor (GR)^9^. It was earlier reported that GR and its transcriptional signaling pathway are activated by shear stress^13^. Consistently, our results showed enhancement of the pFAK level, which is known to be increased by shear stress through the integrin signaling pathway^14^ in osteoclasts, suggesting that osteoclasts were regulated by shear stress-like force specified under microgravity. Although genetic studies have mainly identified FKBP5 as playing a role in depressive illness^18^, and an *in vitro* study showed that FKBP5 overexpression in osteoclasts induces up-regulation of bone resorption and that the *fkbp5* gene expression level in osteoclasts is increased by glucocorticoid treatment^19^. Regarding the role of DDIT4 in bone biology, it was reported that hypoxia-inducible factors including DDIT4 regulate osteoclast-mediated bone resorption^20^; and in a study on for bone substrate-related bone resorption by osteoclasts, the gene expression levels of *fkbp9* and *ddit4* were found to be up-regulated in osteoclasts^21^. Furthermore, various forms of stress induce FKBP5 or DDIT4^22^,^23^. Taken together, our results suggest that, under microgravity in spaceflight, the change in microenvironment specifically around bones where osteoclasts are in close proximity induces morphological alteration of osteoclast mitochondria, which activates the GR pathway controlling FKBP5 and DDIT4 expression for enhancement of osteoclast multi-nucleation. In the future, we hope to perform again space experiments to clarify the mechanism in more detail.

Medaka fish were filmed for abnormal behaviors to consider a physiological change under microgravity. The movies showed that the fish became accustomed to life under microgravity by displaying unique behaviors such as upside-down, vertical, and tight-circle swimming. In addition, we found that the mating behavior at day 33 under microgravity was not different from that on the earth, indicating that the medaka fish had adapted to their microgravity environment. We found a motionless behavior at day 47, an interesting phenotype of adaptation under microgravity. In fact, spaceflight poses chronic stress to animals, which impairs physiological function; and it reduces mechanical use, inducing hypodynamia and hypokinesia. Such effects suggest the presence of some main factor leading to bone loss under microgravity, which situation is similar to disuse osteoporosis on the ground.

Taken together, our results indicate impaired physiological function and reduced mechanical use under microgravity, as well as osteoclast activation as an effect of stress specified by microgravity. The results of our present study provide a better understanding of the response of bone to reduced gravity and shed light on the mechanism underlying the regulation of bone physiology under microgravity.

## Methods

### Medaka Fish

Cab, an inbred wild-type strain of the medaka (O. latipes), was used for this study. The experiments were performed in accordance with policies and protocols approved by the Japan Aerospace Exploration Agency (JAXA) Institutional Animal Care and Use Committee. The fish were kept under a photoperiod of 14-h light/10-h darkness at 28 °C. Eggs were obtained by random crossing and kept at the same temperature just indicated. Embryos were incubated at 28 °C after collection. After hatching, larvae were incubated at room temperature. Earlier we established double transgenic medaka by mating a TRAP-GFP line for visualizing osteoclasts^24^ with an Osterix-DsRed line for visualizing osteoblasts^25^,^26^ and utilized the heterozygous medaka of this double transgenic line.

The medaka fish were flown on a Space-shuttle in 1994, mated, and maintained for 15 days in the second International Microgravity Laboratory^27^. As for medaka osteoclasts, we previously reported that multinucleate osteoclasts are localized at the region of the pharyngeal teeth^8^, in which osteoclasts are visualized by using a TRAP promoter-GFP transgenic line for studying bone resorption during bone remodeling^24^. Furthermore, osteoblasts are visualized by using an Osterix promoter-DsRed transgenic line^25^.

### Selection of medaka

Totally 312 medaka of the TRAP-GFP/Osterix-DsRed double transgenic line with a similar level of growth, confirmed with reference to scale growth, were carried into the Baikonur Space Station and reared up to a total length of 16 mm from the tip of the head to the tip of the caudal fin. During this time, we checked the growth level in terms of total length, and kept those fish with higher growth as a group in the same tank. Finally, we examined the sight of medaka by using a rotation apparatus, with the sight being evaluated by 4 judges who classified the medaka into 4 grades of sight level. Among the fish (6 weeks after hatching), 16 were chosen for long-term rearing in the International Space Station (ISS) and 8 others were additionally chosen as an mRNA source just after arriving at the ISS. On Oct 23 of 2012, Soyuz (assigned as 32S) was launched at 10:51 (GMT) from Baikonur.

### Optomotor response test

The visual test was performed according to an earlier flight experiment using medaka fish^27^. The fish were placed in a circular tank with a rotating wall painted with vertical stripes of black and white colors. When the wall rotates, the fish have a tendency to follow the stripes and thus swim around the tank. Fish with good vision keep swimming around the tank even when the wall rotates very quickly, whereas fish with a poor vision soon see the rotating stripes as a blur and stop moving^28^.

### Rearing system

The aquatic habitat was developed by the JAXA based on the aquatic animal facilities for Space Shuttle use^29^,^30^,^31^. This aquatic habitat is used to rear small freshwater fish such as medaka or zebrafish up to 90 days in space. The habitat was composed of a closed water circulation system that included a water circulation unit, gas exchanger for exchange of dissolved gas in the circulation water, a biological filter for water purification, and 2 aquariums as habitats for the fish. The water circulation unit included water pumps, flow sensors, a temperature controller, and an accumulator to control the environment for fish habitation. The total water volume of the water circulation system was 3.2 L, and the water volume of each aquarium was 0.7 L. The aquarium had an automatic feeder to supply artificial powdery food to the fish. Also, each aquarium was equipped with an LED light for producing day and night cycles and a CCD camera for observation of the fish. Outlines of the aquatic habitat and a flow schematic are shown in the JAXA homepage^32^. These components were assembled in the Multi-purpose Small Payload Rack (MSPR) in the Japanese Experiment Module (JEM) in ISS.

During fish habitation in the aquatic habitat, the water temperature was kept at 25.5–26 °C, and the water flow rate was controlled at 0.1 L per a minute for each aquarium. The dissolved oxygen was maintained at over 90% saturation by gas exchange with the cabin, and the water quality was controlled by using a biological filter of nitrifying bacteria. The water quality was checked by the astronauts once or twice in a week by using test strips, and there was no accumulation of either ammonia or nitrite during the experiments. The day and night cycle, obtained by use of a white LED light, was controlled to be 14 hrs as light (380 lux) and 10 hrs as dim light (10 lux; this dim light was used for dorsal light response of fish). Three kinds of powdery food, Otohime B-1 (Marubeni Nisshin Feed) and Kyowa N250 and N400 (Kyowa Hakko Bio), were supplied to the fish by an automatic feeder in the aquarium twice or 3 times a day.

### Sample transport and fixation condition

Medaka fish were placed in a “Fish Carrier” and flown on Soyuz flight TMA-06M (Roscosmos, Russia). After arrival at KIBO, the space laboratory of Japan in the International Space Station (ISS), the medaka were transported to the “Aquatic Habitat (AQH)” in the “Multipurpose Small Payload Rack (MSPR)” for on board rearing. With consideration of the dorsal light response of fish, the lighting was achieved from the upper side in the tank. The feed was automatically dispensed from the underside of the tank. A filter was used to clarify the water automatically. Subsequently, at selected times fish were caught by a “Fish Catcher” and transported into the “Fish Fixation Apparatus” for analysis of histology or mRNA expression.

Initially, 8 fish were reared in each aquarium. Fourteen days after the start of rearing, 6 fish were anesthetized with ethyl 3-aminobenzoate methanesulfonate salt (tricaine) and fixed with PFA (paraformaldehyde), washed 3 days later, and then stored at 4 °C in a refrigerator in ISS. These 6 sampled fish were brought to the earth by Soyuz on Nov. 19 of 2012. Fifty-six days after the start of rearing, another 6 fish were sampled for PFA fixation, washed 4 days later, and then stored at 4 °C. In addition, 60 days after rearing had begun, the remaining 4 fish were treated with RNAlater (Sigma-Aldrich, MO, USA), and stored at −95 °C in ISS. After the return to earth, PFA-fixed specimens were rinsed several times and stored in cacodylate buffer. These specimens were maintained at 4 °C or −95 °C throughout experiments and transited from Moscow to Japan. Under vivarium conditions, the same numbers of fish were sampled on the ground as control samples in the same time-course experiment. This flight-synchronous (control) group was kept under conditions identical to those of the flight group except for spaceflight.

### Monitoring of fish behavior

For comparison of the behavior between the ground control and flight medaka, movies were recorded 2 or 3 times each day by use of a CCD camera during the 60-day experiment.

### Soft X-ray

Whole medaka fish were subjected to soft X-ray imaging (Sofron sro-M50, Sofron Co., ltd. Tokyo. Japan). The intensity of the white signal was measured by use of software (ImageJ). The gray values of the pharyngeal bone region were corrected by the background of the inner head region.

### Radiological assessments of the pharyngeal bone and vertebrae

Three-dimensional (3D) reconstruction images of pharyngeal bones and vertebrae were obtained by using image analysis software (TRI/3D-view, Ratoc System Engineering, Tokyo, Japan) to analyze the data obtained by micro-focal computed tomography (μ-CT; ScanXmate-E090 [147 μA, 48 kV]; Comscan, Kanagawa, Japan)^33^,^34^. Some of the pharyngeal bone samples were analyzed as higher-resolution images obtained by using a SCANCO Medical μCT 35 System (145 μA, 55 kV; Nippon Denshi-Scanco Medical, Brüttisellen, Switzerland) having an isotropic voxel size of 3.5 μm^35^. The image slices were reconstructed and analyzed for obtaining the focal average thickness by using Scanco μCT Version 6.1 software (Nippon Denshi-Scanco Medical). The bone mineral density (BMD) of the whole pharyngeal bone and vertebrae was measured by use of peripheral quantitative computed tomography (pQCT; XCT Research SA+; Collimation B, Stratec Medizintechnik GmbH, Pforzheim, Germany). We set up a new standard for measuring the BMD of medaka vertebrae, since their mineral content is much lower than that of rodent bones; and, therefore the standard value of 690 mg/cm^3^ for separating cortical bones from trabecular bones could not be used. The mineral bone density was recognized as a bone segment having a focal bone mineral density of more than 100 mg/cm^3^.

In the statistical analysis, all data were presented as the means ± standard deviation (SD). Statistical comparisons were performed by use of the unpaired t-test. The difference was considered significant at p < 0.05.

### Whole-tissue imaging for measurement of cell volume

The specimens fixed with PFA were equilibrated with 80% glycerol. The fluorescent images were collected by a laser-scanning confocal microscope (Olympus, Center Valley, PA; FV1000). The interval of all continuous images was 2 μm in the pharyngeal bone and vertebral body. The range of each tissue was adjusted enough to contain the fluorescence of tissues. The upper pharyngeal bones at day 56 were covered with 130 slices; and the lower pharyngeal teeth and bone at day 56, with 140 slices. The vertebral body at day 56 was covered with 90 slices. The volumes of GFP and DsRed expressing cells were calculated by use of Volocity Demo (Perkin Elmer). We avoided the pigment fluorescent signals as the background.

### Technovit sections for TRAP staining and von Kossa staining

After completion of CLSM examination, a pair of lower pharyngeal bones with attached teeth was collected from each medaka and divided into left and right halves. These specimens were dehydrated through an ascending series of ethanol, embedded in Technovit 7100 (Heraeus Kulzer GmBH, Wehrheim/Ts., Germany), and polymerized at ice-cold temperature. Cured Technovit blocks of lower pharyngeal bones and teeth were serially cut into 4-μm-thick sections and processed for von Kossa staining^36^. The sections were further stained for histochemical demonstration of tartrate-resistant acid phosphatase (TRAP) activity by the Azo-dye method at pH 5.8 (30–45 min at 37 °C). Standardized digital photomicrographs of pharyngeal bones and teeth in serial sections were collected, and the surface areas (pixel numbers) of von Kossa-positive structures (Kossa area) and TRAP-positive structures (TRAP area) were respectively counted by using a computer software (Photoshop 7.0 or CS5 Extended, Adobe, USA). The pixel ratio of Kossa/TRAP areas was calculated for the group reared for 56 days. In the group reared for 14 days, on the other hand, the pixel ratio of TRAP area/whole unit area of measurement was calculated. The pharyngeal bones and teeth of normal medaka, maintained through identical time schedules and feeding conditions under normal gravity were similarly processed and examined as above and served as the control. von Kossa-positive mineralized structures and TRAP-positive osteoclasts were reconstructed into 3D images by using a computer software, DeltaViewer^37^.

### Histology of paraffin sections

One half of a pair of upper pharyngeal bone and teeth from each fish was decalcified in neutralized 10% ethylenediaminetetraacetic acid (EDTA) solution for 1 week at 4 °C prior to paraffin embedding and sectioning.

For immunohistochemistry, the following primary antibodies were used: goat anti-GFP Abs (FITC-labeled) at 1:1000 (abcam, ab6662), rabbit anti-DsRed Abs at 1:1000 (Living Colors, 632496) and rabbit anti-pFAK Abs at 1:200 (Invitrogen, 44624G). The secondary antibodies used were goat anti-rabbit Cy-3 Abs at 1:1000 (Jackson ImmunoResearch 711-165-152).

The sections were reacted with primary Abs overnight at 4 °C after preventing non-specific binding with 2% BSA/TBS and then incubated with secondary Abs and DAPI 30 min at room temperature. The fluorescent images were collected by a laser-scanning confocal microscope (Olympus, Center Valley, PA; FV1000).

The standardized digital photomicrographs of pharyngeal bones and teeth in serial sections were collected, and the area of fluorescence (pixel numbers) and fluorescence intensity (mean value) of anti-GFP, anti-DsRed, and anti-pFAK Abs-reactive regions were respectively determined by using a computer software (Photoshop CS4 or ImageJ). We eliminated DsRed-positive signals in tooth buds from total DsRed signals, so that we used DsRed signals in only pharyngeal bones in the 56-day reared group.

For visualization of collagen matrix, the paraffin sections were stained with the reagents of an Azan staining Kit (Muto Pure Chemicals Co., ltd.) according to the manufacturer’s instructions. The blue area in a randomly selected tooth germ was determined by using computer software (Photoshop CS4).

### Whole-transcriptome analysis

cDNA sequencing was performed for whole-transcriptome analysis (Takara bio). A cDNA library was constructed by using a TruSeq RNA Sample Preparation Kit v2 (illuminakk.co.jp). Double-stranded cDNAs were synthesized by using fragments of PolyA^+^RNA as templates. The sequence data was analyzed with an HiSeq system (illuminakk.co.jp) using a sequence library.

### Semi-quantitative RT-PCR analysis

Total RNA was extracted from jaws preserved with RNAlater according to the manufacturer’s instructions, and cDNAs were synthesized by using a PrimeScript RT reagent Kit with gDNA Erase (TaKaRa). RT-PCR analysis was performed by using EX Taq (TAKARA) according to the manufacturer’s instructions. The sequences of the primers were as follow: *fkbp5*f, TGTCCAGGGTTTGATGTGGA; *fkbp5*r, GTTCTTTGCGTTCCCGACTG; *ddit4*f, GTCCAACAATGGCAGCAGAA; *ddit4*r, TTCCTCCAGTGGGTCAAAGAA; *gapdh*f, CTTGGATACACAGAGGACCAGG; and *gapdh*r, GCCAAACTCATTATCGTACCAT.

## Additional Information

**How to cite this article**: Chatani, M. *et al.* Microgravity promotes osteoclast activity in medaka fish reared at the international space station. *Sci. Rep.* **5**, 14172; doi: 10.1038/srep14172 (2015).

## Supplementary Material

Supplementary Information

Supplementary Movie 1

Supplementary Movie 2

Supplementary Movie 3

Supplementary Movie 4

Supplementary Movie 5

Supplementary Movie 6

Supplementary Movie 7
