# Supplementary material for: Microgravity promotes osteoclast activity in medaka fish reared at the international space station
Source: Sci Rep. 2015 Sep 21;5:14172. doi: 10.1038/srep14172 (PMC4585676; doi:10.1038/srep14172)
Supplement: Supplementary Information [file srep14172-s1.pdf]

Microgravity promotes osteoclast activity in medaka fish reared at the international space station

Masahiro Chatani,[1] Akiko Mantoku,[1] Kazuhiro Takeyama,[1] Dawud Abduweri,[2] Yasutaka Sugamori,[3] Kazuhiro Aoki,[3] Keiichi Ohya,[3] Hiromi Suzuki,[4] Satoko Uchida,[4] Toru Sakimura,[4] Yasushi Kono,[5] Fumiaki Tanigaki,[6] Masaki Shirakawa,[6] Yoshiro Takano,[2] and Akira Kudo[1]

[1] Graduate School of Bioscience and Biotechnology, Tokyo Institute of Technology, Yokohama 226-8501, Japan

[2] Section of Biostructural Science, Graduate School of Medical and Dental Sciences, Tokyo Medical and Dental University, Tokyo 113-8549, Japan

[3] Section of Pharmacology, Department of Bio-Matrix, Graduate School, Tokyo Medical and Dental University, Tokyo 113-8549, Japan

[4] Department of Science and Applications, Japan Space Forum, Tsukuba 305-8505, Japan

[5] Mitsubishi Heavy Industries, Ltd. Kobe 652-8585, Japan

[6] Japan Aerospace Exploration Agency, Tsukuba 305-8505, Japan

Corresponding author:

Akira Kudo, PhD

Department of Biological Information, Tokyo Institute of Technology

4259-B-33 Nagatsuta, Midori-ku, Yokohama 226-8501, Japan

Tel: 81-45-924-5718, Fax: 81-45-924-5718, E-mail: akudo@bio.titech.ac.jp

Running title: Activation of medaka osteoclasts in space

Supplementary Information

Supplementary Figure 1 | Number of pharyngeal teeth at days 14 and 56.

(**A**) Comparison of the number of teeth in ground and flight groups at day 14. Ground n=5, flight n=4. (**B**) Comparison of the number of teeth in ground and flight groups at day 56. Ground n=6, flight n=5.

Supplementary Figure 2 | Soft X-ray analysis of day 14 medaka shows no significant decrease in the mineral density around the pharyngeal region.

(**A**) Lateral views of head region of ground-(left) and flight-group (right) medaka at day 14. Scale bar indicates 2 mm. (**B**) Comparison of "mean gray value" of white signals in the pharyngeal region between ground and flight groups.

Supplementary Figure 3 | Radiological assessment of the vertebral bone in 56-day flight group.

(**A**) Bone mineral density (BMD) of vertebrae measured by pQCT. (**B**) There was no significant difference in the bone mineral density between the ground and flight groups; Ground n=6, Flight n=5.

Supplementary Figure 4 | Fluorescence imaging of day-56 vertebral body shows an increase in the ratio of GFP volume/DsRed volume in the flight group.

(**A**) Lateral views of fluorescent images for ground-group (left) and flight-group (right) vertebral body at day 56. (**B**) Quantitative analysis of volume of GFP fluorescence. Ground (left), flight (middle), mean (right). (**C**) Quantitative analysis of volume of DsRed fluorescence. Ground (left), flight (middle), mean (right). (**D)** Ratio of GFP

volume/DsRed volume. Ground n=5, flight n=5.

Supplementary Figure 5 | Antibody staining for GFP and DsRed in day-14 fish

(**A**) Double staining with anti-GFP and anti-DsRed Abs of ground-group medaka (left) and flight-group ones (right) at day14. Scale bar indicates 100 µm. (**B**) Comparison between the ground and flight groups for GFP-positive area per DsRed area. (**C**) Comparison between the ground and flight groups for the signal intensity of GFP per DsRed. Ten paraffin sections of the pharyngeal bone region per medaka at day 14 were selected from ground (n=3) and flight (n=3) groups. *P<0.05, by Student's *t*-test. Error bars, s.e.m.

Supplementary Figure 6 | Histochemical analysis of TRAP enzyme reactions in osteoclasts associated with pharyngeal bones and teeth at day 14.

(**A-D**) Medaka from the ground group at day 14. (**E-H**) Fish from the flight group at day 14. Four-µm-thick Technovit sections incubated for TRAP reactions were counter-stained with methylene blue or toluidine blue. Rectangles in "D" and "H" delineate unit areas (186 x 280 μm^2) for which the percentage of TRAP-positive areas was calculated in each sample. Arrows indicate the rostral direction; and scale bars, 200 µm. (**I**) Percentage of TRAP-positive osteoclast area in 186 x 280 μm^2 unit area of 20 randomly selected sections (5 sections per medaka) from the ground- and flight-group sections shown in "A"-"H." Proportion of TRAP-positive area is significantly larger in the flight group. **P<0.005, by Student's *t*-test. Error bars, s.e.m.

Supplementary Figure 7 | Reconstructed 3D images of TRAP-stained cells from

day-14 medaka.

(**A-D**) TRAP enzyme reactions in osteoclasts at day 14, shown in red color, are associated with lower pharyngeal bones and teeth from ground medaka (A, B) and flight medaka (C, D). The arrow indicates the rostal direction. (**E**, **F**) Spatial relation between TRAP-positive osteoclasts (OC) and pharyngeal epithelial components (green) in the lower pharyngeal tooth-forming region of a day-14 flight medaka viewed from 2 different angles. Bones and teeth are excluded. The arrow indicates the rostral direction. TG, tooth germ.

Supplementary Figure 8 | 3D imaging of GFP area as a whole in day-56 medaka lower pharyngeal bone.

Oblique images were reconstructed by using the images collected by confocal laser scanning. Comparison of TRAP-GFP-positive cell volume between the ground group (left) and flight group (right) at day 56. The results of quantitative analysis of GFP volume are shown in Fig. 3d. Bars represent 400 μm.

Supplementary Figure 9 | Altered mitochondrial morphology in flight-group jaw.

Electron microscopic analysis of mitochondria in osteoclasts in ground control (A-D) and flight (E-H) medaka. (**A, B, E, F**) Selected areas with mitochondria colored in red. (**C, D, G, H**) Each figure shows the extracted red color region corresponding to mitochondria shown in “A,” “B,” “E,” and “F.” (**I**) Comparison of red color area between ground control and flight groups, showing no significant difference between them. (**J**) Comparison of roundness measurement (circularity) between ground control and flight groups, showing a reduction in the roundness ratio of the flight group. In the

control group, a total of 140 mitochondria depicted from 3 osteoclasts was measured; while in the flight group, 214 mitochondria from 7 osteoclasts were evaluated. **P<0.005, by Student's *t*-test. Error bars, s.e.m. [circularity = 4π (area/perimeter squared)]

Supplementary Figure 10 | Fluorescence imaging of osteoclasts and osteoblasts in the jaw.

(**A, B**) Frontal view of jaw opening of day-14 ground medaka (A) and day-14 flight medaka (B) showing fluorescence of TRAP-GFP-expressing osteoclasts (green) and Osterix-DsRed-expressing osteoblasts (red) in the upper and lower jaws. Whole-mount preparations are shown.

Supplementary Figure 11 | Decrease in the amount of collagen matrix in the flight group.

(**A**) Azan-stained pharyngeal bone region of ground (left) and flight (right) groups. Blue color indicates the collagen-rich connective tissue area. Arrowheads point to tooth germs. Asterisks show bones degraded by osteoclasts from the anterior side. Rostral direction is to the left. Scale bar indicates 50 μm. (**B**) Comparison of the collagen-rich area in tooth germs between ground and flight groups. Collagen-rich areas in 49 and 23 pharyngeal tooth germs were randomly selected from 21 and 21 paraffin sections from day-56 ground-group medaka (n=3) and day-56 flight-group medaka (n=3), respectively. The collagen-rich area was slightly decreased in the latter group. Error bars, s.e.m.

Supplementary Figure 12 | Transmission electron micrographs (TEM) of

osteoblasts in the jaw bone of day-56 ground-group medaka.
Osteoblasts enriched with rough endoplasmic reticulum are seen. There was no significant difference in cytoplasmic features between the ground (upper) and flight (lower) groups. OB: osteoblast.

Supplementary Figure 13 | Image of a full-length agarose gel used for RT-PCR analyses.
“G” indicates the ground control; and “F,” the flight medaka. The image of a full-length agarose gel for RT-PCR analyses of *fkbp5* and *ddit4* is shown, while the same data are shown in Figure 5A as the image of a cropped gel. The *gapdh* is the control.

Supplementary Table 1 | Preparation of medaka at Bikonur and experiments at ISS.
The flight fish were prepared for the space experiment on the ground at Baikonur. Afterwards, the ground-control specimens were prepared under the same conditions.

Supplementary Table 2 | Total number of nuclei in GFP-positive cells.
Table shows the number of GFP-positive cells containing each number of nuclei. Comparison between the ground and flight groups as to the total number of nuclei in GFP-positive cells. GFP-positive areas in 30 serial paraffin sections of pharyngeal bone region were selected from ground medaka (n=3) and flight medaka (n=3) at day 56. The graph was shown in “Fig. 3N.”

Supplementary Table 3 | Altered levels of gene expression following 60-day

spaceflight.

(**A**) Changes of >9.0-fold in glucocorticoid-related gene expression in jaw tissues from flight samples compared with gene expression in ground-control samples (P < 0.05). The expressions of *fkbp5* and *ddit4* were strongly increased in the flight-group jaw. Interestingly, *fkbp5* and *ddit4* were enhanced in other tissues including scales and a part of the body. (**B**) Changes giving a >1.2-fold increase in mitochondrion-related gene expression in jaw tissues from flight samples compared with gene expression in ground-control samples (P < 0.05). (**C**) Changes resulting in a >1.4-fold increase in osteoclast-related gene expression in jaw tissues from flight samples compared with gene expression in ground-control samples (P < 0.05). (**D**) Changes giving a <1.5-fold decrease in osteoblast-related gene expression in jaw tissues from flight samples compared with gene expression in ground-control samples (P < 0.05).

Supplementary Movie 1 | Behavioral adaptation at day 0.

At day 0, immediately after transport to the AQH, fish under microgravity swam gently and seldom showed “looping behavior,” which is indicated by swimming in tight circles.

Supplementary Movie 2 | Eating behavior at day 14.

Compared with those at day 0, fish in ground and flight groups swam actively at day14. Interestingly, only the flight fish in microgravity showed a unique swimming style that involved upside-down, vertical, and tight-circle swimming. The feed was automatically dispensed from the underside of the tank 15 seconds after the start of this movie.

Supplementary Movie 3 | Mating behavior under microgravity.

At day 27, fish in the flight or ground group were swimming while holding eggs, suggesting that these fish had mated successfully. This movie shows the mating behavior in the flight medaka at day 33. Male fish turned to position themselves under the female fish at 7 seconds after the start of this movie and “held” the female at 9 seconds. Afterwards, they were moving to the bottom during 16-20 seconds. Regardless of the low gravity, this mating behavior showed the same pattern as seen for the medaka on the earth.

Supplementary Movie 4 | Hypokinetic behavior in the flight group at day 47.

The fish tended to become motionless in the late stage, suggesting hypokinesia.

Supplementary Movie 5 | 3D imaging of anti-GFP/anti-DsRed immunostaining of pharyngeal bone.

Partial 3D models of osteoclasts and osteoblasts from the ground (left) and flight (right) pharyngeal bone/teeth at day 56, as reconstructed from 5-μm-thick paraffin sections that had been doubly stained with anti-GFP and anti-DsRed Abs. The intensity of the anti-GFP FITC Ab signals was increased in the flight group.

Supplementary Movie 6 | Magnified view of 3D imaging of anti-GFP/anti-DsRed immunostaining for pharyngeal bone.

Magnified partial 3D models of osteoclasts and osteoblast from the ground (left) and flight (right) pharyngeal bone/teeth at day 56 reconstructed from 5-μm-thick paraffin sections that were doubly stained for anti-GFP and anti-DsRed Abs. The anti-GFP FITC

Abs signals were increased in flight.

Supplementary Movie 7 | 3D imaging of TRAP/von Kossa staining in day-56 medaka.

Partial 3D models of pharyngeal bones/teeth and osteoclasts from the ground (left) and flight (right) at day 56 reconstructed from 4-μm-thick Technovit sections that were doubly stained for von Kossa and TRAP reactions. The gap in the von Kossa staining implies possible dissolution of minerals from bones and teeth in the flight medaka.

Supplementary Fig. 1

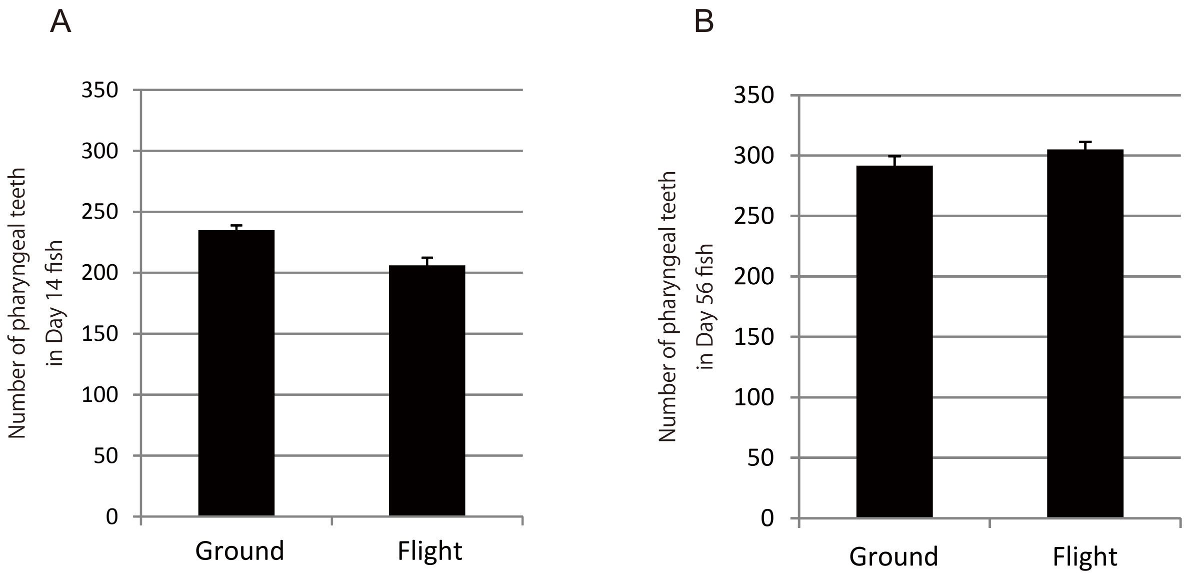

Supplementary Fig. 2

A

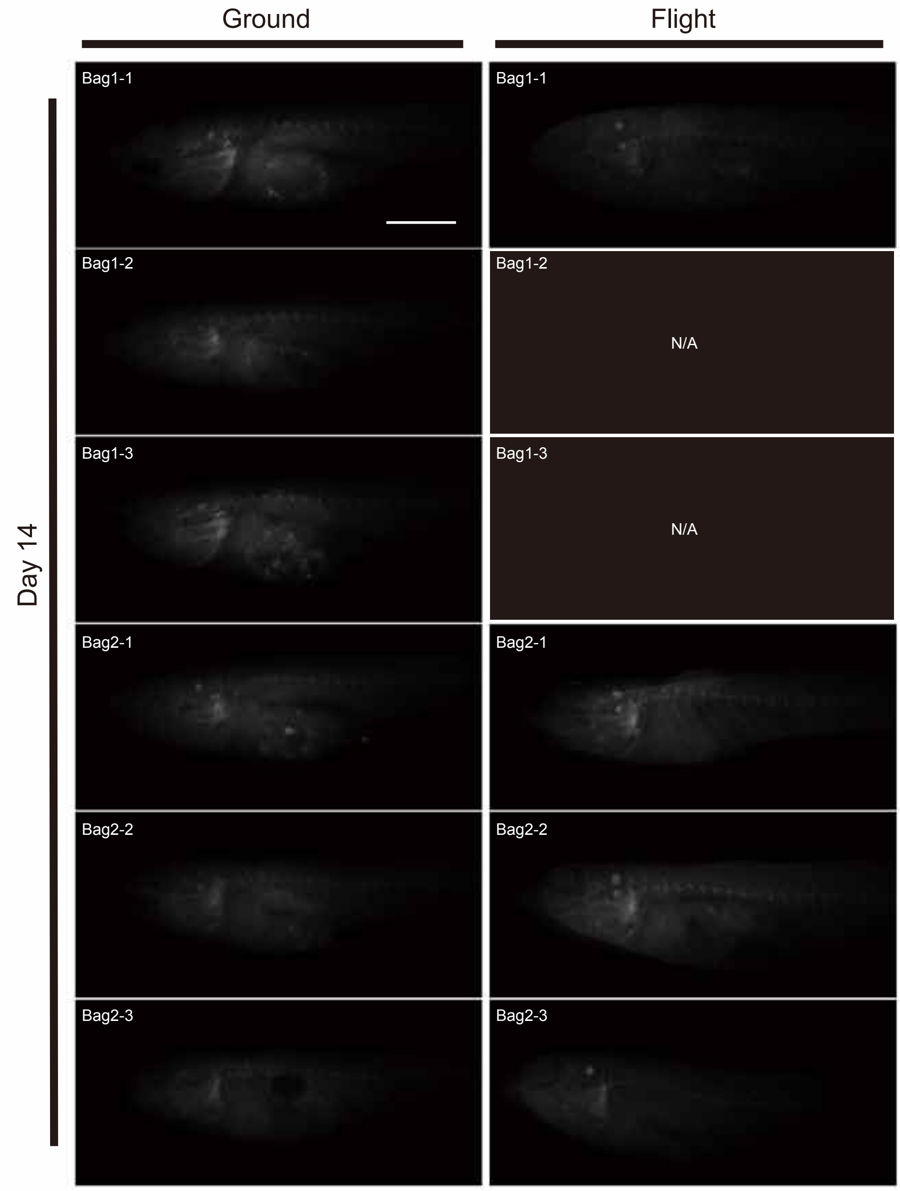

B

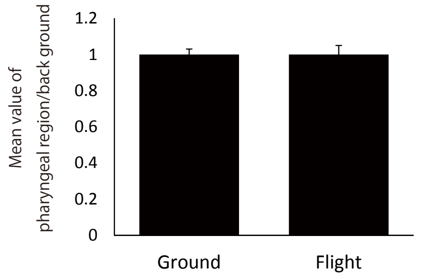

Supplementary Fig. 3

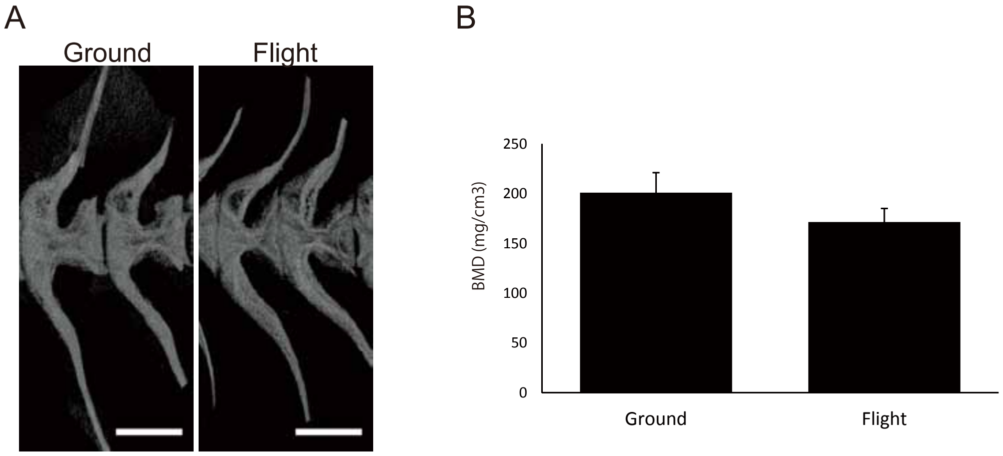

Supplementary Fig. 4

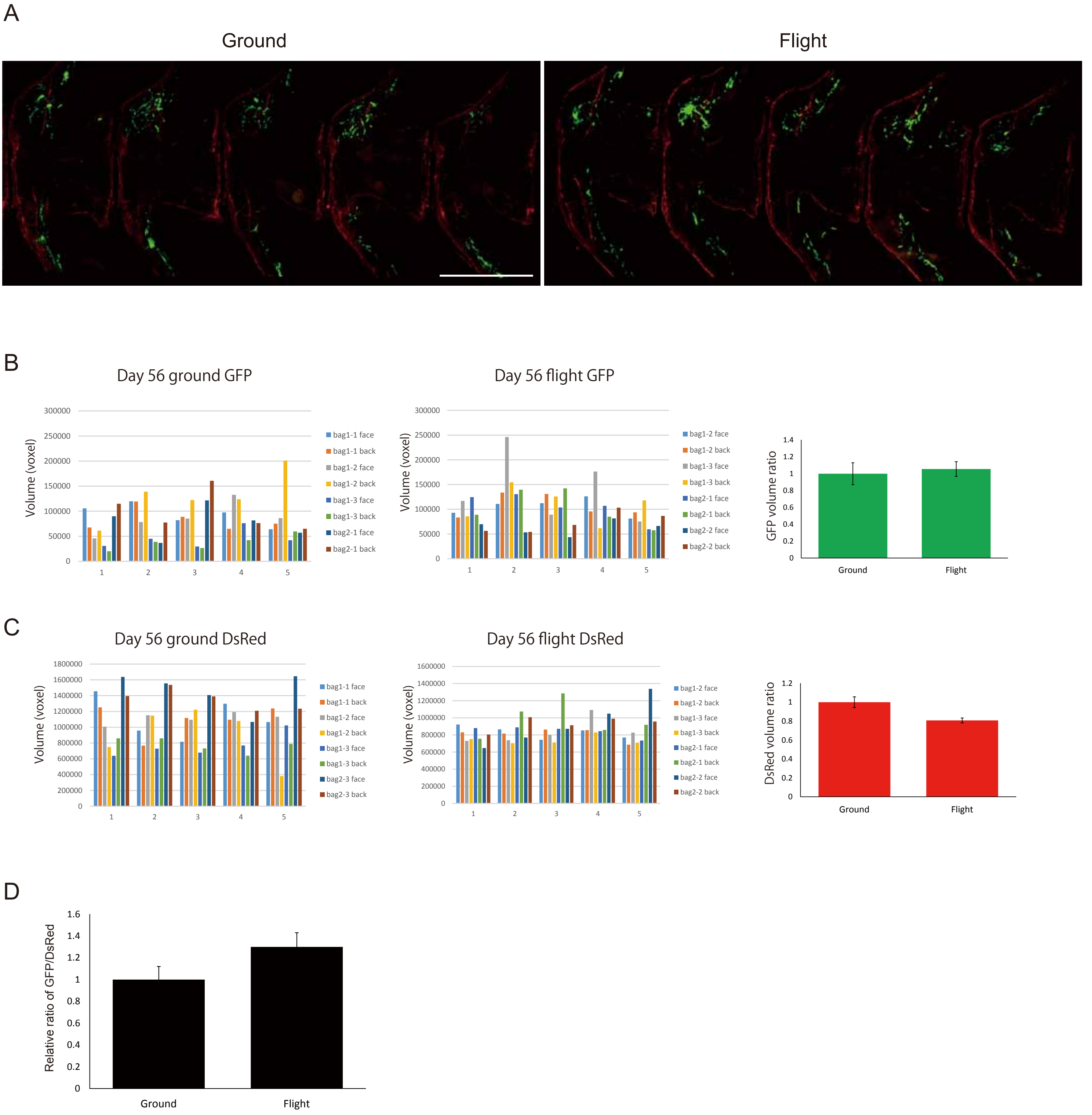

Supplementary Fig. 5

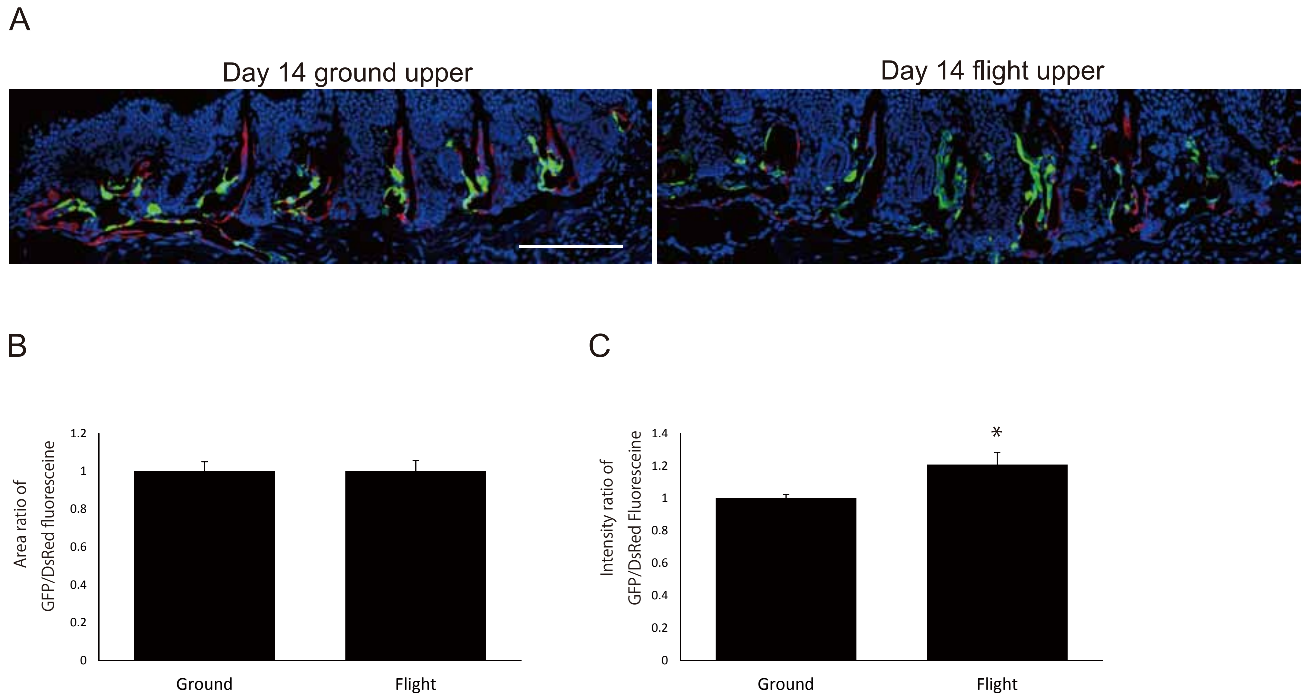

Supplementary Fig. 6

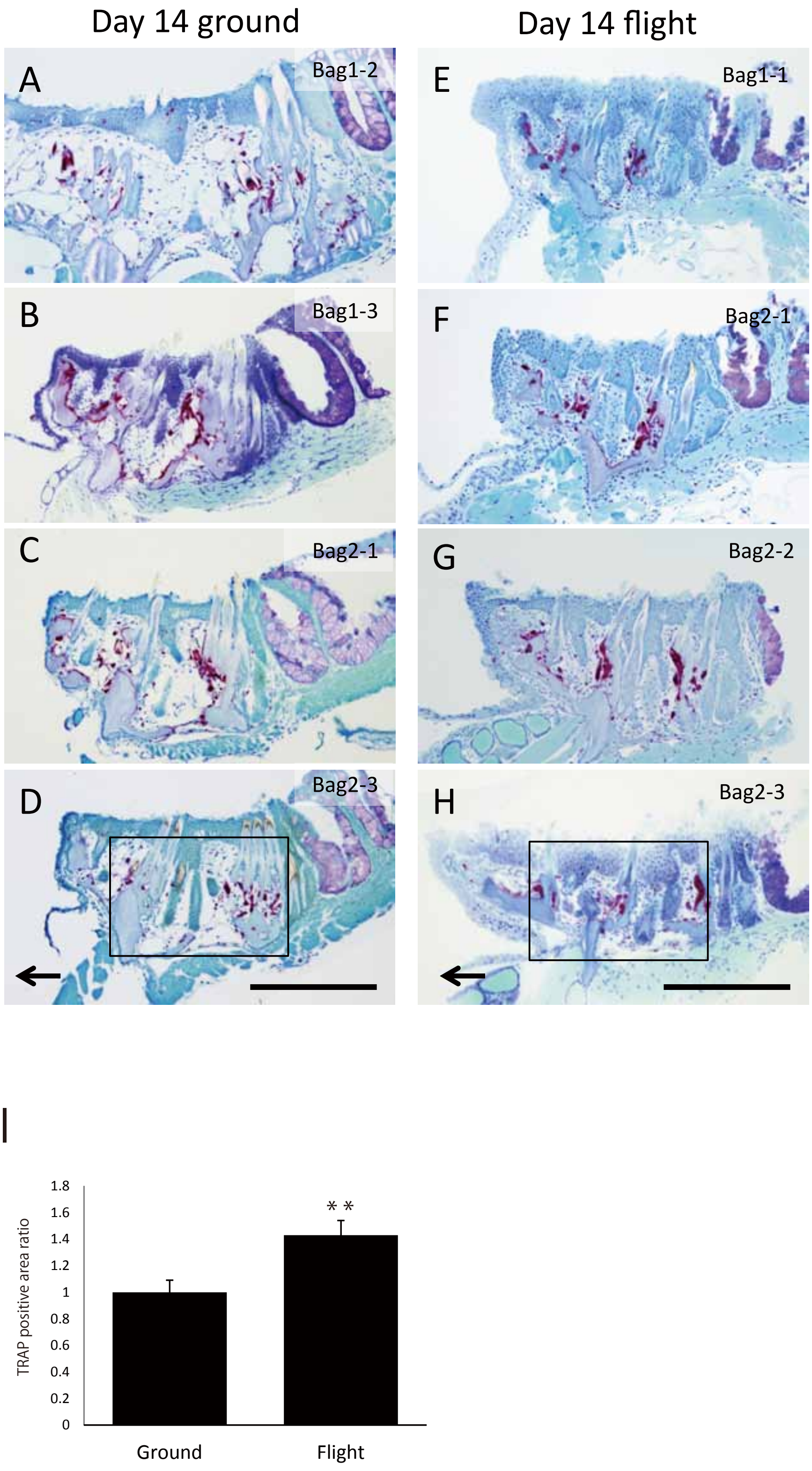

Supplementary Fig. 7

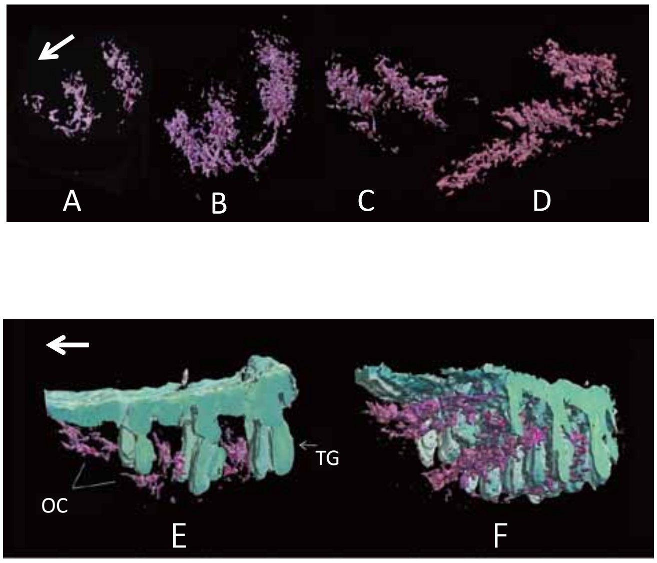

Supplementary Fig. 8

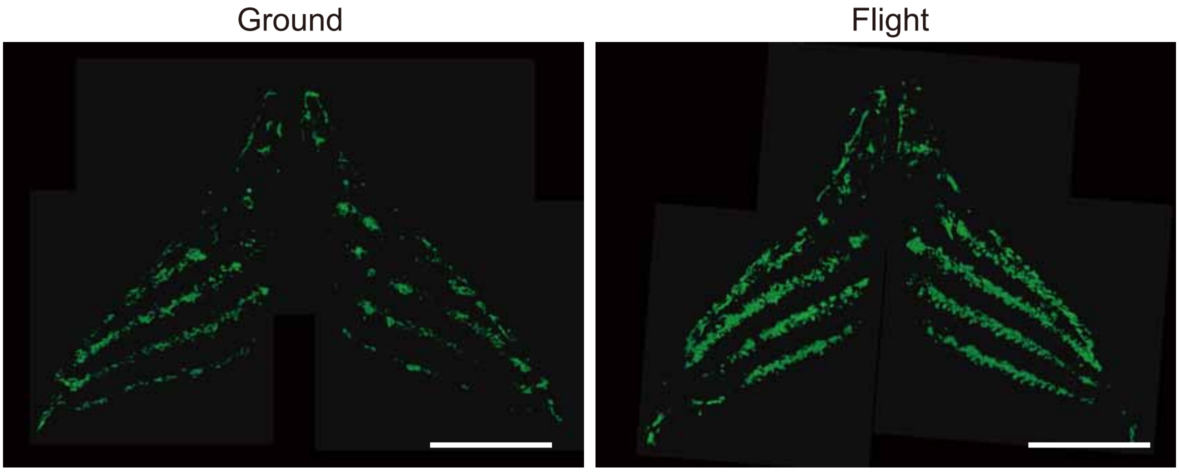

Supplementary Fig. 9

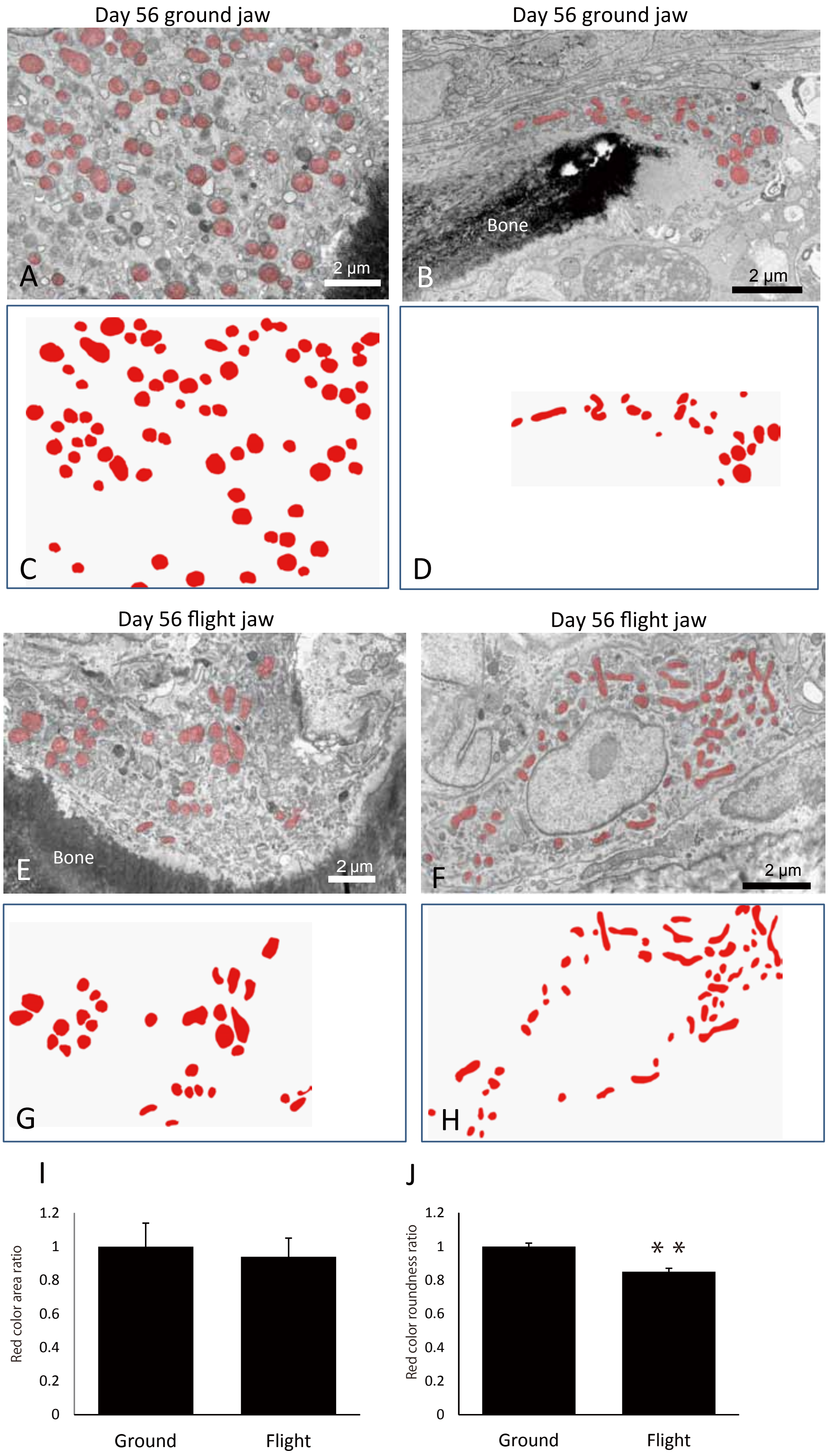

Supplementary Fig. 10

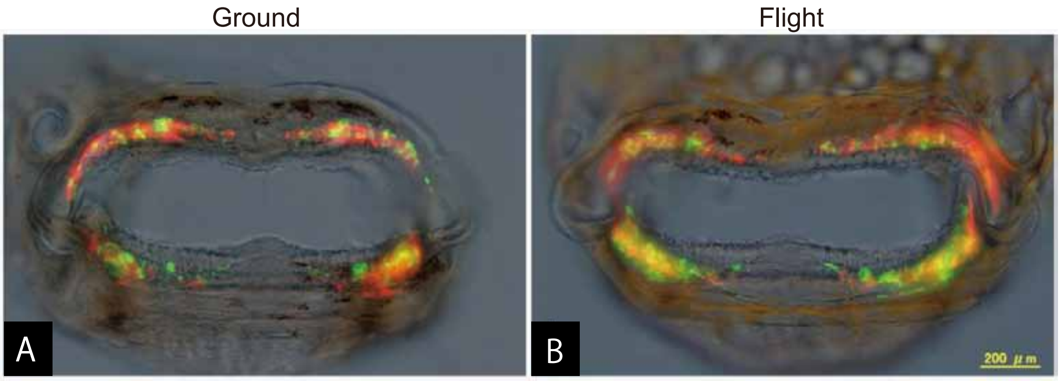

Supplementary Fig. 11

A

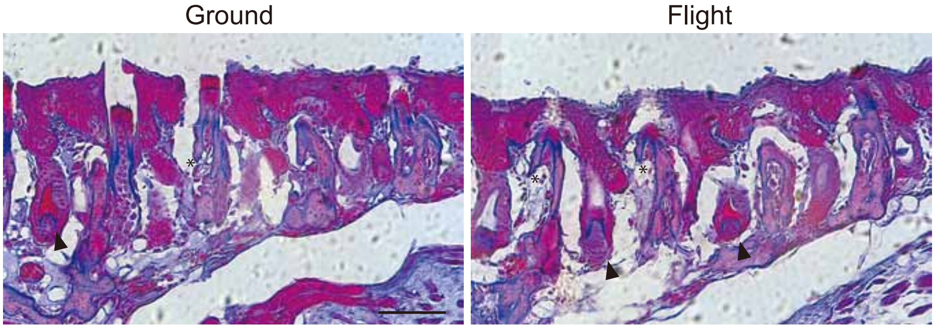

B

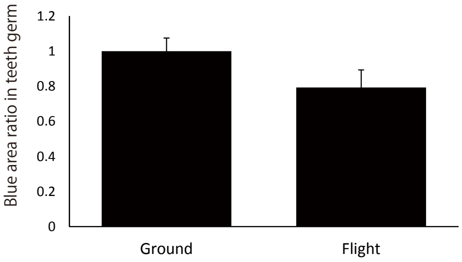

Supplementary Fig. 12

Day 56 ground jaw

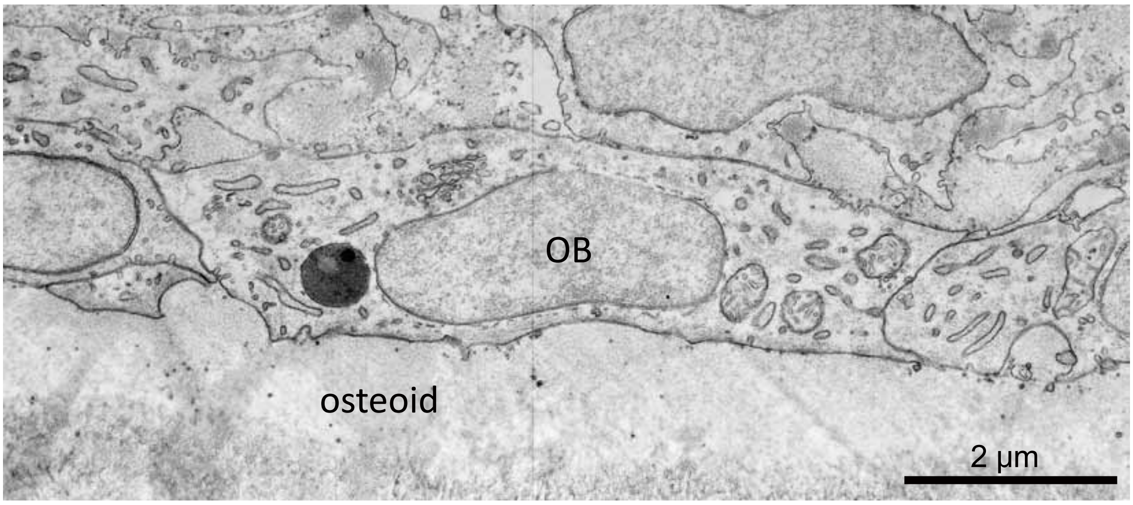

Day 56 flight jaw

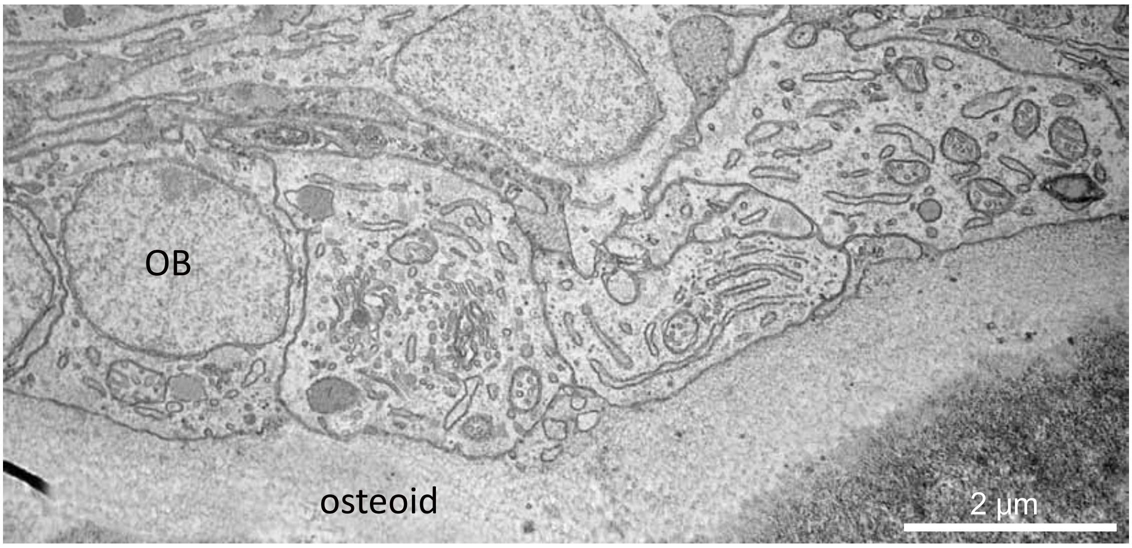

Supplementary Fig. 13

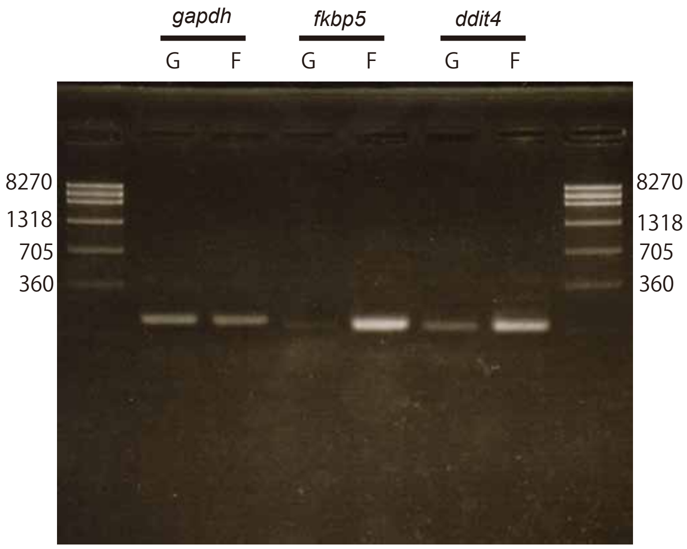

Supplementary Table 1

Preparation of medaka at Bikonur and experiments at ISS

Date (GMT)	Time course	Event
23-Oct-12	18hrs before launch	Preparation of fish for launch
23-Oct-12	Launch at 10:51	Launch of Soyuz
25-Oct-12	2 days after launch	Docking of Soyuz to ISS
25-Oct-12	8 hrs after docking	The start of experiment in AQH
25-Oct-12	10 hrs after docking	*8 fish were fixed with RNAlater
08-Nov-12	14 days after the start of experiment	6 fish were fixed with PFA
20-Dec-12	56 days after the start of experiment	6 fish were fixed with PFA
24-Dec-12	60 days after the start of experiment	4 fish were fixed with RNAlater

* Manuscript in preparation

Supplementary Table 2

Number of nuclei		1	2	3	4	5	6	7	8	9	10	11	12	13
The number of GFP-cell	Ground	490	188	73	24	16	7	6	0	1	0	1	0	0
	Flight	383	165	62	27	25	11	4	6	1	5	2	0	2

Supplementary Table 3

Type	Gene name	Fold change	Style	Ground RPKM	Flight RPKM	Accession no.
a	fkbp5	26.4	up	10.6	280.1	ENSORLG00000020069
	ddit4	12.5	up	11.1	138.3	ENSORLG00000009171
b	phosphoenolpyruvate carboxykinase 2 (mitochondrial)	3.7	up	10.4	37.9	ENSORLG00000006281
	isocitrate dehydrogenase 2 (NADP+), mitochondrial	2	up	3.1	6.2	ENSORLG00000012601
	cytochrome c oxidase subunit 6A, mitochondrial	1.6	up	36.8	59	ENSORLG00000002089
	creatine kinase, mitochondrial 2 (sarcomeric)	1.6	up	71.2	110.2	ENSORLG00000000769
	tspo (translocator protein)	1.5	up	39.3	57.7	ENSORLG00000019914
	cyc1 (cytochrome c)	1.4	up	76.5	104.8	ENSORLG00000020555
	ATP synthase F0 subunit 6 (mitochondrion)	1.4	up	2253.5	3096.2	ENSORLG00000020024
	mitochondrially encoded cytochrome b	1.3	up	2460.1	3300.5	ENSORLG00000021782
	mitochondrially encoded NADH dehydrogenase 4	1.3	up	718.5	946.6	ENSORLG00000021775
	mitochondrially encoded NADH dehydrogenase 6	1.3	up	304	397.1	ENSORLG00000021780
	translocase of outer mitochondrial membrane 20 homolog (yeast)	1.3	up	25.5	32.04	ENSORLG00000001214
	mitochondrially encoded cytochrome c oxidase II	1.2	up	229.3	283	ENSORLG00000021766
	mitochondrially encoded cytochrome c oxidase III	1.2	up	4809.8	5905.8	ENSORLG00000021770
	isocitrate dehydrogenase 2 (NADP+), mitochondrial	1.2	up	179.6	223.6	ENSORLG00000008218
	mitochondrially encoded NADH dehydrogenase 4L	1.2	up	671.1	827.8	ENSORLG00000021774
c	cebpb	2.7	up	240.4	647.9	ENSORLG00000015032
	fosl1a	2.6	up	31.5	81.5	ENSORLG00000002045
	fosb	2.1	up	121.5	260.5	ENSORLG00000006665
	c-fos	1.6	up	479.7	780.8	ENSORLG00000017502
	junbb	1.5	up	362	535	ENSORLG00000015530
d	osterix	0.6	down	12.5	7.2	ENSORLG00000005215
	col1a2 (collagen, type I, alpha 2)	0.7	down	1454	987.7	ENSORLG00000004319
	col1a1b (collagen, type I, alpha 1b)	0.7	down	1151.1	777.9	ENSORLG00000011273
	col1 (collagen type I alpha 1)	0.7	down	1308.8	932.9	ENSORLG00000017013
	col10a1 (collagen, type X, alpha 1)	0.7	down	1015.2	732.6	ENSORLG00000013436
	cbfa1	0.9	down	11.5	10.3	ENSORLG00000010169
	alpl (alkaline phosphatase-like)	0.9	down	14	12.8	ENSORLG00000017066
